# Empowering Families Through Technology: A Mobile-Health Project to Reduce the TAND Identification and Treatment Gap (TANDem)

**DOI:** 10.3389/fpsyt.2022.834628

**Published:** 2022-02-28

**Authors:** Tosca-Marie Heunis, Stacey Bissell, Anna W. Byars, Jamie K. Capal, Nola Chambers, Sebastián Cukier, Peter E. Davis, Liesbeth De Waele, Jennifer Flinn, Sugnet Gardner-Lubbe, Tanjala Gipson, J. Christopher Kingswood, Darcy A. Krueger, Aubrey J. Kumm, Mustafa Sahin, Eva Schoeters, Catherine Smith, Shoba Srivastava, Megumi Takei, Stephanie Vanclooster, Agnies M. van Eeghen, Robert Waltereit, Anna C. Jansen, Petrus J. de Vries

**Affiliations:** ^1^Mental Health and Wellbeing Research Group, Department of Public Health, Vrije Universiteit Brussel, Brussels, Belgium; ^2^Cerebra Network for Neurodevelopmental Disorders, University of Birmingham, Birmingham, United Kingdom; ^3^Division of Neurology, Department of Pediatrics, Cincinnati Children's Hospital Medical Center/University of Cincinnati College of Medicine, Cincinnati, OH, United States; ^4^Department of Neurology, University of North Carolina at Chapel Hill, Chapel Hill, NC, United States; ^5^Centre for Autism Research in Africa (CARA), Division of Child and Adolescent Psychiatry, University of Cape Town, Cape Town, South Africa; ^6^Argentine Program for Children, Adolescents and Adults With Autism Spectrum Disorders (PANAACEA), Buenos Aires, Argentina; ^7^Department of Neurology, Boston Children's Hospital and Harvard Medical School, Boston, MA, United States; ^8^Department of Paediatric Neurology, University Hospitals Leuven, Leuven, Belgium; ^9^Department of Development and Regeneration, KU Leuven, Leuven, Belgium; ^10^TSC Canada, Mississauga, ON, Canada; ^11^Department of Statistics and Actuarial Sciences, Stellenbosch University, Stellenbosch, South Africa; ^12^Department of Pediatrics, University of Tennessee Health Sciences Center, Memphis, TN, United States; ^13^Le Bonheur Children's Hospital and Boling Center for Developmental Disabilities, Memphis, TN, United States; ^14^Department of Clinical Genetics, St George's University Hospitals, London, United Kingdom; ^15^Sussex Renal Unit, The Royal Sussex County Hospital, Brighton, United Kingdom; ^16^TSC Clinic Cincinnati Children's Hospital Medical Center, Cincinnati, OH, United States; ^17^Clinical Pediatrics and Neurology, Department of Pediatrics, University of Cincinnati College of Medicine, Cincinnati, OH, United States; ^18^Rosamund Stone Zander Translational Neuroscience Center, Boston Children's Hospital, Boston, MA, United States; ^19^Be-TSC, Mortsel, Belgium; ^20^TSCi, Mortsel, Belgium; ^21^TSC Alliance, Silver Spring, MD, United States; ^22^Tuberous Sclerosis Alliance India, Mumbai, India; ^23^Japanese Society of Tuberous Sclerosis Complex, Family Network, Tokyo, Japan; ^24^Emma Children's Hospital, Amsterdam University Medical Center, Amsterdam, Netherlands; ^25^TAND Expert Centre, ‘s Heeren Loo, Hoofddorp, Netherlands; ^26^Child and Adolescent Psychiatry, University Medical Center Göttingen, Göttingen, Germany; ^27^Pediatric Neurology Unit, Department of Pediatrics, Antwerp University Hospital, Antwerp, Belgium

**Keywords:** tuberous sclerosis complex, TSC-associated neuropsychiatric disorders (TAND), digital technology, health app, personalised medicine, rare diseases, behavioural phenotypes

## Abstract

**Introduction:**

Tuberous Sclerosis Complex (TSC) is a multi-system genetic disorder with various TSC-Associated Neuropsychiatric Disorders (TAND) that significantly impact the mental health and wellbeing of individuals with TSC and their caregivers. TAND represents the number one concern to families worldwide, yet is highly under-identified and under-treated. The clinician-administered TAND-Checklist (Lifetime version, TAND-L) has improved identification of TAND in clinical settings. However, many individuals with TSC and their caregivers still have difficulty accessing suitable support for diagnosis and evidence-informed interventions. The TANDem study is a community-based participatory research project with a broad range of TSC stakeholders aimed at reducing the TAND identification and treatment gap.

**Objectives:**

Participatory research identified three priority next steps: 1) development and validation of a self-report, quantified version of the TAND Checklist (TAND-SQ) and building the TAND-SQ into a smartphone application, 2) generation of consensus clinical recommendations for the identification and treatment of TAND, to be incorporated as a TAND toolkit on the app, and 3) establishment of a global TAND consortium through networking, capacity-building and public engagement activities.

**Methods:**

TANDem is a four-year project, and includes 24 consortium members from 10 countries representing all World Health Organization regions. Collaborators represent five stakeholder groups (family representatives, technology experts, clinical experts, non-profit organisations and researchers). Here we outline the project study protocol in detail, describing the scientific rationale, the project aims and objectives, the methods involved in participant recruitment, multi-site and multi-phase data collection, data analysis, ethical considerations including informed consent, data protection, privacy and confidentiality considerations related to the European Union General Data Protection Regulation and the USA Health Insurance Portability and Accountability Act. The expected outcomes and potential impact on the TSC community, implementation and dissemination of results, as well as future scale-up and scale-out plans are also discussed.

**Conclusions:**

The TANDem project has the potential to transform the global TSC community by empowering families living with TSC through an easily accessible digital solution to allow them to document their own TAND needs linked to an evidence-informed toolkit to enhance personalised healthcare, and by providing healthcare professionals with consensus clinical recommendations to prevent, identify and manage TAND manifestations.

## Introduction

### Background

Tuberous Sclerosis Complex (TSC) is a rare genetic disease with multi-system manifestations including the brain, kidneys, skin, heart, and lungs (1). It has a birth incidence of approximately 1 in 5,000 to 10,000 live births (2, 3). Many have suggested that the prevalence of TSC has been underestimated due to factors such as under-recognition of less severe phenotypes, marked variability of symptoms and lack of genetic testing (4, 5). Appropriate management and coordination of medical specialist care are crucial throughout the lifespan of individuals with TSC to reduce morbidity and mortality (6, 7). Overall, clinical care has improved significantly over the last two decades, and clinical trials and evidence-based guidelines for many of the physical manifestations have led to better outcomes worldwide (7). TSC is also associated with a significant range of neuropsychiatric manifestations including developmental disorders (such as autism and intellectual disability), mental health problems (such as anxiety and mood disorders), scholastic difficulties, and specific neuropsychological deficits (1, 8). These neuropsychiatric manifestations lead to the greatest burden of care to families and are strongly related to psychosocial problems and family stress. Unfortunately, these are also the manifestations that are typically not identified and treated in TSC (1, 8–10).

In an attempt to raise awareness, to outline the multi-level manifestations, and to develop a ‘shared language' for these neuropsychiatric manifestations we coined the term ‘TAND' (TSC-Associated Neuropsychiatric Disorders) and recommended annual screening for TAND (7, 8). In response to the need of the TSC community, we next developed and pilot validated the TAND Checklist, a simple and free pen-and-paper checklist to guide conversations about TAND between clinicians and families (8, 11). The current version of the TAND Checklist (TAND Checklist-Lifetime version, TAND-L) has been implemented widely in the TSC community. At least 19 language translations of the TAND Checklist have been authorised to date and are available free-of-charge (www.tandconsortium.org) (8, 9). Unfortunately, the findings from the large-scale TOSCA natural history study of TSC showed that TAND manifestations were often not identified and psychiatric disorders were under-diagnosed or diagnosed late, even in expert TSC centres around the globe (10). Even though coining of the term ‘TAND' and the development of the TAND Checklist have helped to raise awareness about TAND, the TOSCA findings therefore underlined the urgency of finding more empowering strategies to identify and treat TAND.

One of the real-life challenges of TAND is the fact that people with TSC seem to have vastly different and unique TAND profiles and, until recently, no data-driven investigations have been able to find any replicable TAND profiles. This observation of course underlines the importance of a personalised approach to treatment of TAND. Unfortunately, however, this ‘overwhelming uniqueness' had also led to ‘treatment paralysis' among clinicians (12). In order to manage the overwhelming uniqueness of individual TAND profiles, we proposed that data-driven methodologies would help us to identify a smaller number of ‘natural TAND clusters.' In this context, a natural cluster is defined as a number of TAND manifestations that typically occur together. In a feasibility study of 69 individuals with TSC across all ages, we used TAND Checklist data and applied a range of cluster and factor analysis methods (13). We identified six natural TAND clusters, all with good face validity, and suggested that data-reduction may be feasible using the TAND Checklist (13). The findings were replicated in a slightly larger international study (14). We next applied comparable methods to an international group of 453 individuals with TSC from six international sites (including Belgium, South Africa, and the USA) and identified seven natural TAND clusters, very similar to the feasibility findings (15). Identified clusters included an autism-related cluster, a dysregulated cluster, an eat/sleep cluster, a mood/anxiety cluster, a neuropsychological cluster, an overactivity/impulsivity cluster, and a scholastic cluster (15). We proposed that these clusters could be used for psychoeducation of families and professionals and to develop tailored approaches to identification and treatment of TAND. In addition, we suggested that the different natural TAND clusters may point to potential differential aetiological underpinnings and responses to molecular and other treatments of TAND manifestations (16).

As part of the natural TAND cluster study, we incorporated focus group interviews with families from all over the globe. Focus groups were conducted in South Africa, USA, Europe and Australia with more than 50 TSC patients, parents/caregivers, family members and professional experts. Research partners included Tuberous Sclerosis International, European Tuberous Sclerosis Association, Tuberous Sclerosis Association (UK) and the TSC Alliance (USA). The main emphasis of the focus groups was to seek perspectives and recommendations from global TSC stakeholders about next step use of the TAND Checklist and natural TAND clusters (10).

Four main themes emerged from qualitative thematic analysis. Firstly, stakeholders felt that the TAND Checklist had provided the TSC community with a powerful tool to identify the range of TAND difficulties experienced by families on a daily basis. However, families expressed concern that the current version of the TAND Checklist was validated as an interview between a clinician and family. There was a clear desire to have a *self-report version* of the TAND Checklist that could be used by families even outside the context of a clinical visit. A number of families described how they had already used the TAND Checklist as a self-help tool to guide understanding of their family member's TAND profile. Secondly, stakeholders reported the desire for a *quantified version* of the TAND Checklist. Families described that the lifetime version of the TAND Checklist (TAND-L) focused on the lifetime occurrence of TAND manifestations. However, families were also keen to evaluate current needs and to quantify these needs, for instance, the severity of symptoms and/or their functional impact on daily life. Thirdly, a request from families was for a *toolkit of advice, information, recommendations, and general intervention strategies* to help them manage the TAND manifestations identified in their family member. This was the strongest and universal theme that emerged from the qualitative findings (‘Can you tell us what we can do about TAND?'). Families described that most of them, regardless of where they were in the world, had struggled to access suitably-qualified therapeutic support, given barriers such as clinical care pathways, referral systems, waiting lists or funding. The fourth theme evident from data was a strong recommendation to use *digital technology* to make the TAND Checklist, quantification, and toolkit available to families across the globe (15).

### Study Rationale, Aims, and Objectives

The aims of the TANDem project were shaped in partnership with the TSC user/caregiver community, and were therefore in direct response to the priority needs of individuals who live with TSC and their families. The need for TAND interventions had been articulated strongly in the TSC community, and recently also through a powerful participatory process funded by the King Baudouin Foundation in Belgium (17). Through a multi-step consensus-building process, a panel of people with lived experience in TSC and a panel of stakeholders with professional expertise in TSC generated 15 priorities for TSC research. In the study, TAND was identified as the most frequently endorsed area of concern. The first item on the priority list for research was the need for interventions for TAND, which will be addressed in the current project. In addition, the TANDem project will directly or indirectly respond to five other identified priorities: reducing the translational gap from research to real-world settings, determining additional evidence to be generated to substantiate treatment recommendations, early identification and treatment of manifestations, considering how (inter)national patient registries can be set up in sustainable ways, and identifying the most appropriate family support to guide acceptance and to mitigate the impact of TSC throughout the lifespan.

Given the significant identification, treatment and research gap for TAND, it was felt appropriate to address the priority requests from families and professional stakeholders through a multi-stakeholder participatory study, using a mixed-method approach.

**Figure 1:**
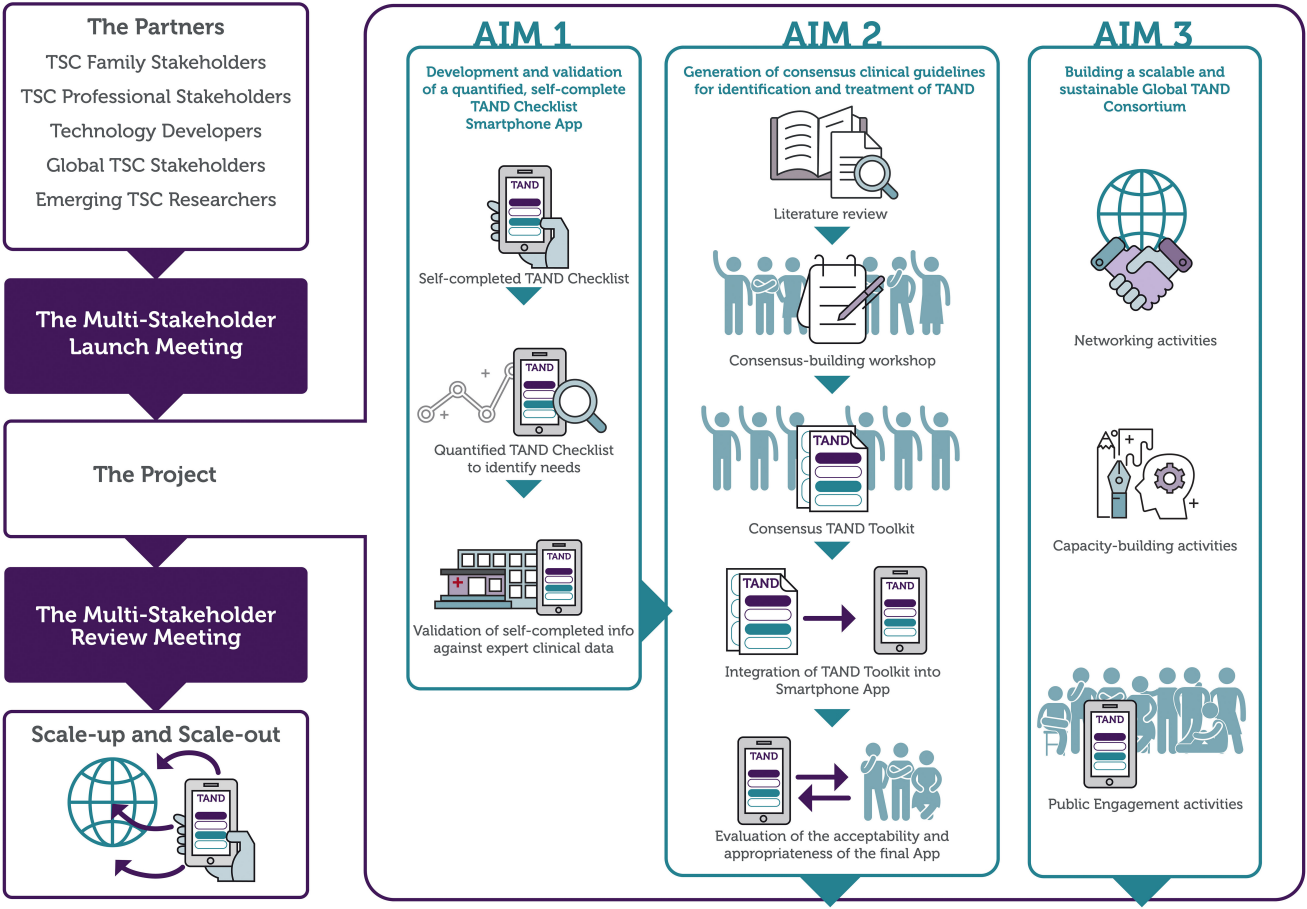
An overview of the TANDem project.

The TANDem project aims and objectives are shown in Table 1. Figure 1 provides a visual overview of the project.

**Table 1:** TANDem project aims and objectives.

Aim 1: To develop and validate a self-report, quantified TAND Checklist (TAND-SQ), and to build it into a smartphone application (app).	Objective 1.1 Generate a self-report TAND Checklist
	Objective 1.2 Quantify the TAND Checklist
	Objective 1.3 Develop a smartphone app based on the TAND-SQ
	Objective 1.4 Validate self-completed TAND app data against expert clinical data
Aim 2: To generate consensus clinical recommendations for the identification and treatment of TAND, and incorporate these into the TAND app.	Objective 2.1 Scoping review of existing literature on interventions for TAND
	Objective 2.2 Generate consensus clinical recommendations for identification and treatment of TAND
	Objective 2.3 Generate a TAND toolkit based on literature and consensus recommendations
	Objective 2.4 Integration of the TAND toolkit into the TAND app
	Objective 2.5 Feasibility evaluation, including acceptability and appropriateness, of the final TAND app
Aim 3: To build a scalable and sustainable global TAND consortium through networking, capacity-building and public engagement activities.	Objective 3.1 Conduct networking activities between all global collaborators
	Objective 3.2 Capacity-building of emerging TAND researchers
	Objective 3.3 Public engagement activities to understand societal perspectives on TSC and TAND and to raise awareness of TAND and TSC
	Objective 3.4 Perform a multi-stakeholder review of the TAND app and integrated toolkit
	Objective 3.5 Plan and coordinate scale-up, scale-out and future TAND research

## Methods

### TAND Consortium and Working Groups

#### TAND Consortium

The TAND consortium includes 24 members from 10 countries, representing all World Health Organization regions. Members include a broad range of stakeholder groups (including family representatives, non-profit organisations, researchers, technology experts, clinicians, social scientists, statisticians) and bring highly interdisciplinary skills to the project (including child and adolescent psychiatry, pediatric neurology, clinical psychology, educational psychology, speech and language therapy, special education, intellectual disability medicine, nephrology, biomedical engineering, biostatistics, veterinary sciences, behavioral sciences, neurosciences, and digital technology expertise).

**Figure 2:**
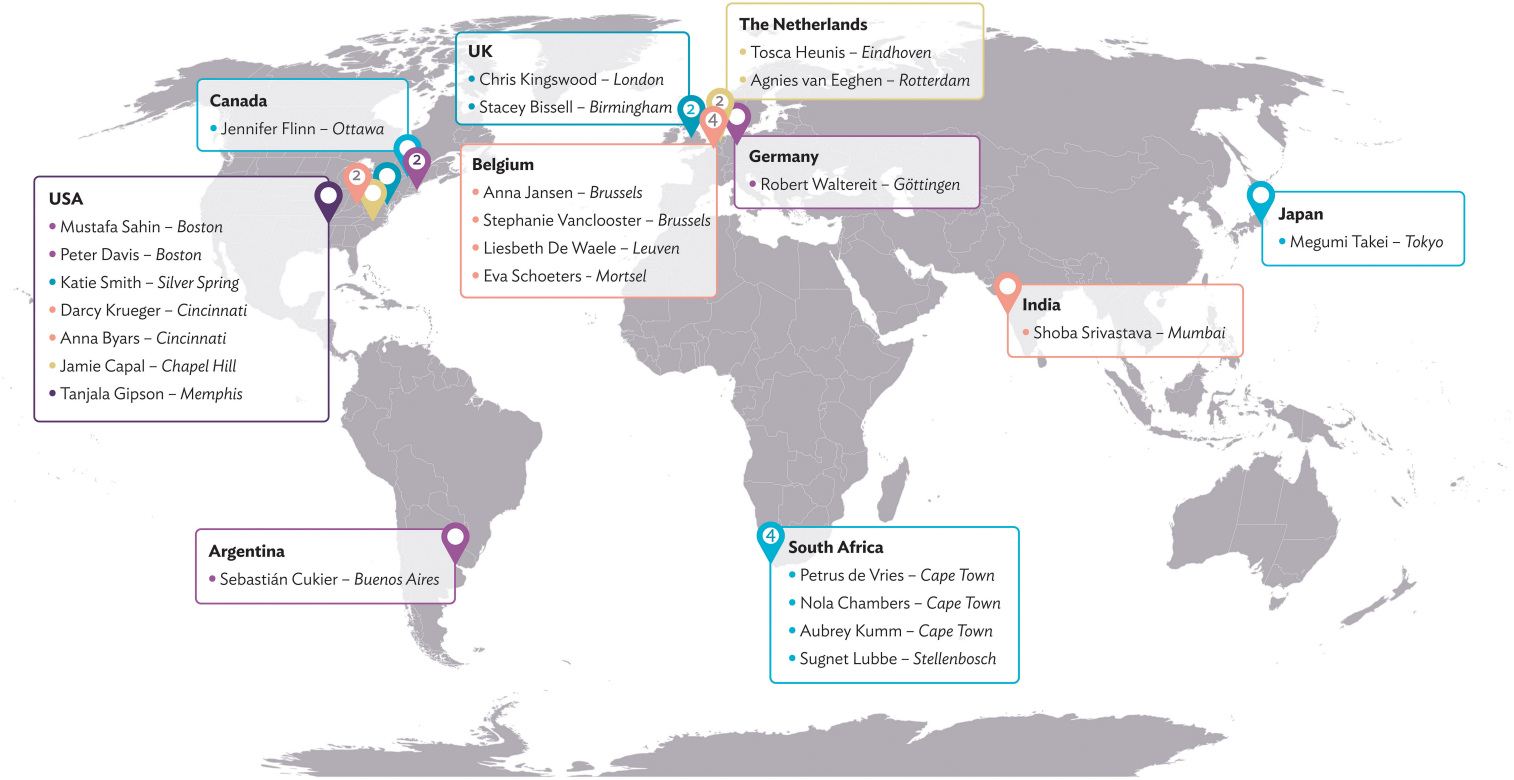
TAND consortium.

**Figure 3:**
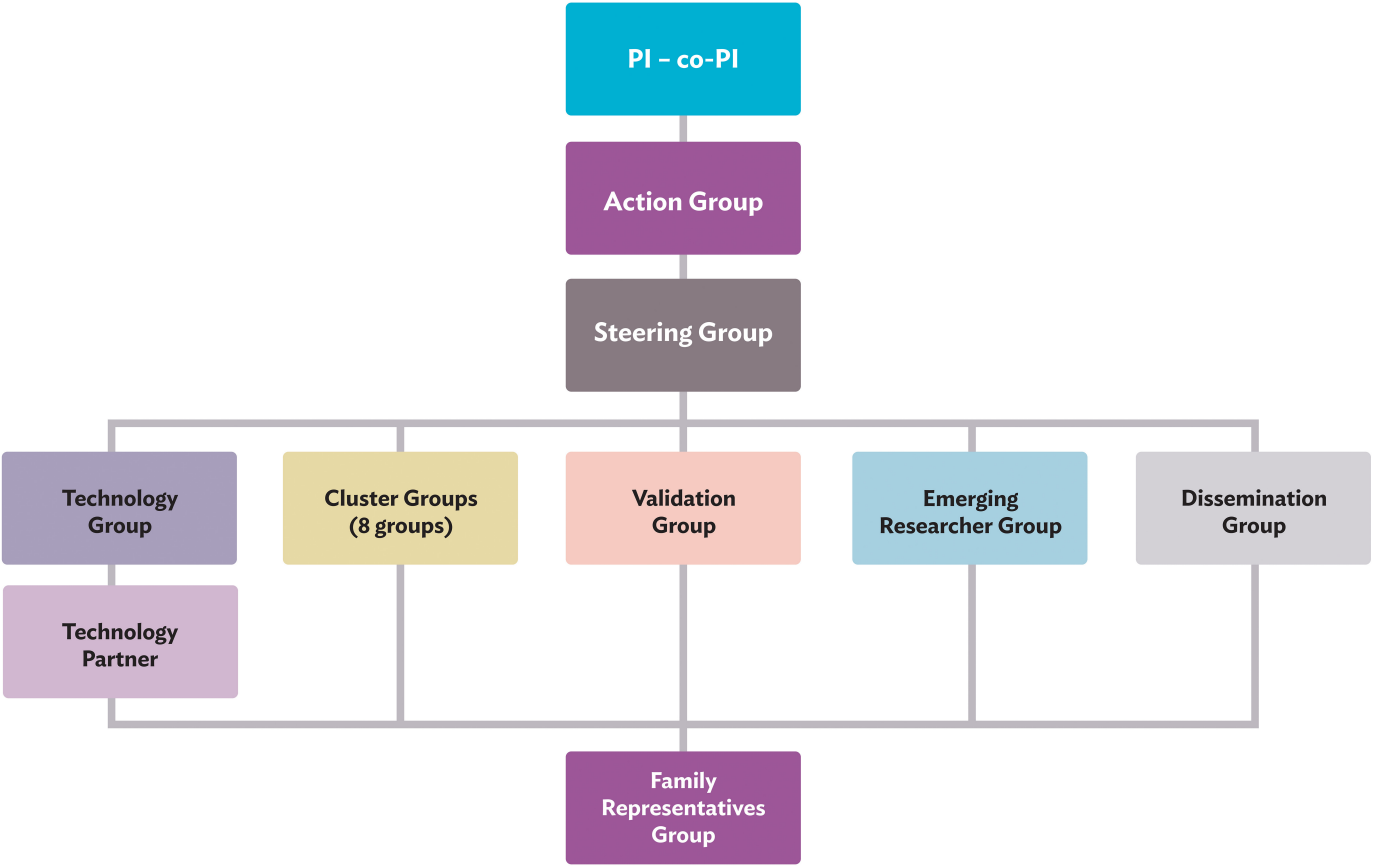
TAND consortium working groups. PI, principal investigator; Co-PI, Co-principal investigator.

This collaboration between individuals with lived expertise and professional expertise leads to cross-fertilisation and new synergies between established and young TSC clinicians and researchers, professionals from outside the TSC community, and family representatives from different parts of the world. The global map in Figure 2 shows all consortium members and their geographical locations. The TAND consortium work together in various working groups, as illustrated in Figure 3 and described under Working Groups.

#### Working Groups

##### Principal Investigator and Co-principal Investigator

The consortium is led by the principal investigator Petrus de Vries and co-principal investigator Anna Jansen who are responsible for the successful running and completion of the project. They are also responsible for all aspects of project oversight, financial management, human resource management, and ethical conduct of the TANDem project.

##### Action Group

The Action Group includes Petrus de Vries, Anna Jansen, Liesbeth De Waele (dissemination lead), Tosca Heunis (project coordinator), and Stephanie Vanclooster (postdoctoral researcher). They are responsible for the day-to-day running of the project and for coordination between all groups in the project. The Action Group meets on a weekly basis to discuss project matters.

##### Steering Group

The Steering Group includes all members of the Action Group as well as Chris Kingswood (family representative and representative of the Tuberous Sclerosis Association UK) and Shoba Srivastava (family representative and member of Tuberous Sclerosis Alliance of India). The Steering Group is responsible for oversight of the activities in the TANDem project and, in particular, provide a forum to bring a user/caregiver voice to the project.

##### Technology Group and Technology Partner

The Technology Group is led by the project coordinator, Tosca Heunis. The group includes Liesbeth De Waele, Chris Kingswood, Aubrey Kumm, and Peter Davis. The Technology Group, in close collaboration with the app development company, is responsible for all aspects related to development of the TAND Toolkit App. The app development requirements are further discussed under App Development.

##### Cluster Groups

The Cluster Groups are overseen by the postdoctoral researcher, Stephanie Vanclooster. Eight cluster groups were created to develop consensus recommendations for the assessment and treatment of TAND in TSC. Each cluster group (see Table 2) has a lead, a co-lead, and additional members, with at least one family representative. Cluster Groups are responsible for conducting a literature review and drafting of a ‘cluster chapter' that focuses on the assessment and treatment of their assigned TAND cluster. These cluster chapters will form the basis of: (1) a book for families and practitioners (most comprehensive), (2) consensus clinical recommendations for TAND (summary of chapters), and (3) a toolkit to be built into the TAND Toolkit App (practical and specific elements from the consensus recommendations). The toolkit development is discussed further under Toolkit Development.

**Table 2:** Cluster groups and members.

Autism spectrum disorder-like cluster	Nola Chambers (lead), Jamie Capal (co-lead), Eva Schoeters, Sebastián Cukier, Shoba Srivastava
Dysregulated behavior cluster	Tanjala Gipson (lead), Peter Davis (co-lead), Agnies van Eeghen
Eat/sleep cluster	Stacey Bissell (lead), Katie Smith (co-lead), Peter Davis
Mood/Anxiety cluster	Agnies van Eeghen (lead), Jamie Capal (co-lead), Megumi Takei, Robert Waltereit
Neuropsychological cluster	Anna Byars (lead), Jennifer Flinn (co-lead)
Overactive/impulsive cluster	Robert Waltereit (lead), Stacey Bissell (co-lead), Katie Smith, Megumi Takei
Psychosocial cluster	Stephanie Vanclooster (lead), Sebastián Cukier (co-lead), Chris Kingswood, Eva Schoeters, Katie Smith,
Scholastic cluster	Jennifer Flinn (lead), Peter Davis (co-lead), Shoba Srivastava

##### Validation Group

The Validation Group is responsible for the validation of the app-based TAND-SQ Checklist (a self-report, quantified version of the TAND-L Checklist) against expert clinical data. It includes the Steering Group along with Mustafa Sahin, Peter Davis, Darcy Krueger and representatives from the three validation sites at Boston Children's Hospital, Cincinnati Children's Hospital, and the TSC Alliance.

##### Emerging Researchers Group

Given that research mentoring and capacity building are goals of the TANDem project, a separate group was set up for self-identified emerging/early career researchers. This group is led by Stephanie Vanclooster. She is joined by Agnies van Eeghen, Peter Davis, Sebastián Cukier, Shoba Srivastava, Stacey Bissell, Tanjala Gipson, and Tosca Heunis. Their meetings are used as a forum to talk about career development, research and mentoring ideas.

##### Dissemination Group

This group is responsible for communication and dissemination of information about TAND and the TANDem project. The project has a website (www.tandconsortium.org), YouTube Channel and Twitter account. This group is led by Liesbeth De Waele and includes the Action Group and various other consortium members (Agnies van Eeghen, Aubrey Kumm, Jamie Capal, Katie Smith, Shoba Srivastava, Stacey Bissell, Sugnet Lubbe, and Tanjala Gipson).

##### Family Representatives Group

The TANDem project was fundamentally set up as a participatory project with multilevel stakeholders. For this reason, it was important to ensure that people with TSC and families who live with TSC have a clear and strong voice at all levels of the project. The Family Representatives Group therefore includes all TANDem members who have a lived experience with TSC: Chris Kingswood, Eva Schoeters, Jennifer Flinn, Megumi Takei, and Shoba Srivastava. Katie Smith, a global Tuberous Sclerosis International representative, is also part of this group. All of them wear multiple ‘hats' as part of the project, either as scientists, educators, clinicians, or as leaders in global non-profit organisations. Family representatives are part of all other groups in the project to ensure that the project will deliver direct benefits to the TSC community.

### App Development

The TAND Toolkit App will allow users to register in a secure way and to add a number of members to their ‘TSC family' profile. Once registered, users will be able to capture a ‘TSC Story' (information about the TSC diagnosis), complete a self-report, quantified version of the TAND Checklist—the TAND-SQ (to identify and quantify the severity of TAND difficulties experienced), view the ‘TAND clusters' profile (that illustrates how different TAND clusters are affected), and to view the ‘TAND toolkit.' The TAND toolkit will provide evidence-informed information about each cluster, including an introduction to the cluster, what families should seek (e.g., assessment, diagnosis, and treatment by a professional), and what families can do themselves (e.g., self-help, home-based actions, strategies and interventions tips). The app also has a functionality to allow ‘surveys' to be done (to collect additional information from users about various aspects of TAND) at any stage during the use of the app. App development was divided into two phases—phase I of the app includes all development except for the toolkit, which forms part of phase II. The app will be accessible on Android and iOS mobile devices.

### Toolkit Development

We will perform a scoping review of all primary literature on interventions for TAND using a standard scoping review methodology (18). The scoping review will serve two purposes: first, to describe the landscape of all TAND research to date in order to identify research gaps in the science of TAND; second, to act as background literature for the formulation of consensus clinical recommendations. In addition, cluster groups will perform expert targeted reviews of the TSC literature and of the broader scientific literature relevant to clusters. Each cluster group will draft a chapter to summarise the background literature and scientific evidence and will generate summary statements and recommendations based on the TAND literature and their expertise. Cluster chapters will be reviewed by two reviewers and presented to the consortium. Cluster chapters will represent a second source of evidence-base towards consensus recommendations development. All cluster summary statements and recommendations will be entered into an online form and consortium members will be asked to rate and rank statements and recommendations. These data will identify items with clear agreement/disagreement, in preparation for face-to-face discussions in a virtual consensus conference. Overall recommendations will be evaluated using the Grading of Recommendations Assessment, Development and Evaluation (GRADE) framework rating tools (19).

Once consensus recommendations have been generated, a process will start to develop the ‘toolkit' to be built into the app. The toolkit will include practical, useful yet evidence-informed information, advice and resources relevant to each cluster. Toolkit development will be supported by a series of ‘creative sessions' with each cluster team to help translate consensus recommendations into app screens. All cluster app screens will be reviewed and revised by cluster teams and the whole consortium. On completion of revised cluster app screens, all information will be shared with the app development team to incorporate into the TAND Toolkit App, phase II of app development.

### Study Design and Implementation

#### Study Design

The TANDem project is a multi-stakeholder participatory project that has included TSC family stakeholders from the project outset—individuals diagnosed with TSC, and parent/caregivers/families of individuals with TSC. As previously mentioned, TSC family stakeholders have already been instrumental in the prioritisation of TAND research goals and in focus group work to seek directions for TAND Checklist research. This ‘lived expertise' input directly led to the project outlined here.

All TSC family stakeholders have participated in the multi-stakeholder launch meeting where the project was discussed in detail. Throughout the 4 years of the project, family stakeholders will participate in achieving the three project aims (see Table 1). In addition, all stakeholders will participate in the review meeting at the end of year three, where the final TAND Toolkit App and the TAND toolkit will be reviewed and where planning for ‘scale-up and scale-out,' and future TAND research will take place.

Family stakeholders and technical partners will be treated equally for their time and input to the project. It should be clear that the philosophy of this project is one of ‘nothing about us without us' (20). We firmly believe that only when ‘lived expertise' and ‘technical expertise' is brought together, truly impactful research in TSC can be conducted.

#### Study Population and Recruitment

The study population of this project consists of children, adolescents and adults diagnosed with TSC and their families (parents/caregivers and other family members). No children below the age of 13, individuals with intellectual disability or other groups of vulnerable participants will be included as direct participants in this study. However, to ensure applicability of the study to these groups, parents/caregivers of younger children and adults with intellectual disability will be invited to participate.

This study will be conducted in partnership with five organisations: Boston Children's Hospital (BCH, USA), Cincinnati Children's Hospital (CCH, USA), the TSC Alliance (USA), Universitair Ziekenhuis Brussel (UZB, Belgium), and Universitaire Ziekenhuizen Leuven (UZL, Belgium).

Recruitment will be done through the primary clinical contacts at the respective sites. The clinicians and their teams who will invite the participants all have expertise in working with patients with TSC, are all familiar with the clinical and personal needs of the families, and are therefore best positioned to determine whether study participation is appropriate. They are also all members of the TAND consortium and familiar with the TAND Checklist under investigation here.

#### Sample Size

The TANDem project will collect data in five steps (as outlined under Data Collection and Analysis). Pre-pilot data steps (1, 2, and 4) will include small sample sizes and will focus on feasibility of measures and methods. Step 3 (validation step) will include TAND-SQ data collection via the app and through a secure electronic portal, and comparison thereof with retrospective expert clinical/research data. Data will be used to replicate and expand previous cluster findings (13, 14, 16) and to examine the sensitivity, specificity and positive predictive value of the TAND-SQ in relation to expert clinical data. There is no consensus in the cluster analysis literature about the ideal sample size. With rare and extremely rare diseases the use of standard statistical methodology for sample size determination is not practical as the condition affects only a very small number of individuals. There is, however, reasonable consensus that a variable to sample ratio of 1:10 is good (i.e., if 10 variables = 100 participants) (13–15). The TANDem project will use 19 variables in analysis, suggesting a sample size of 190 participants. Given that we will have two different ‘kinds' of data for validation (app data and portal data), the final sample size was set at *n* = 100 (portal data linked to real world clinical data), and *n* = 100 (app data linked to detailed research phenotyping data). Step 5, feasibility evaluation of the TAND Toolkit App, will be performed as a mixed-methods step with some quantitative data, but will predominantly involve qualitative data collection through individual interviews and/or focus group discussions. The sample size for this step was set at n = 40. We hope to collect data on a larger sample, but acknowledge the logistical, data transfer and ethical challenges of data collection across international borders.

#### Data Collection and Analysis

##### Overview of Study Steps

There are five steps of data collection and analysis involved, as outlined in Table 3. This table provides a summary of the participants, data collection sites, data to be collected, and data analysis to be conducted.

**Table 3:** Steps of data collection and analysis.

**Step**	**Description**	**Participants**	**Data collection**	**Data analysis**
Step 1	TAND-SQ pre-pilot study	± 20 participants ± 20 TAND consortium members	Each participant and consortium member will complete a paper TAND-SQ and a checklist feedback form.	Mixed-methods analysis of feasibility data. This will inform final TAND-SQ design.
Step 2	App phase I pre-pilot study	± 20 TAND consortium members	Each consortium member will conduct user acceptance testing of phase I of the app, and complete an app feedback form.	Mixed-methods analysis of app feedback data. This will inform final app design before validation (step 3).
Step 3	TAND-SQ validation study	± 100 participants from BCH ± 100 participants from CCH ± 100 participants from the TSC Alliance	BCH and CCH participants will complete a TSC Story, the TAND-SQ Checklist, and an app feedback form. TSC Alliance participants will complete the TAND-SQ Checklist via a secure electronic portal.	BCH/CCH data will be used to evaluate the external and predictive validity of the TAND-SQ by comparing app data to detailed phenotypic data collected as part of a TSC research project (21). TSC Alliance data will be used to evaluate the external and predictive validity of the TAND-SQ by comparing self-reported TAND-SQ data through the online portal with real-world clinical TAND data, also collected through the portal. All TAND-SQ data will be used to replicate and extend previous cluster findings. All feedback form data will be used for descriptive and summative analysis.
Step 4	App phase II pre-pilot study	± 20 TAND consortium members	Each consortium member will conduct user acceptance testing of phase II of the app, and complete a toolkit feedback form.	Mixed-methods analysis of toolkit feedback data. This will inform final app design before feasibility evaluation.
Step 5	App feasibility evaluation study	± 40 participants from BCH, CCH, TSC Alliance, UZB, UZL	Each participant will conduct user acceptance testing of the full app (phases I and II), complete an app feedback form, a toolkit feedback form, and participate in a focus group/semi-structured interview.	Mixed-methods analysis of app and toolkit feedback data and framework analysis of qualitative data.

*BCH, Boston Children's Hospital; CCH, Cincinnati Children's Hospital; UZB, Universitair Ziekenhuis Brussel; UZL, Universitaire Ziekenhuizen Leuven*.

##### Step 1: The TAND-SQ Pre-pilot Study

Each participant and consortium member will be invited to complete a paper TAND-SQ Checklist, and to share their feedback via a checklist feedback form. Data will be used to perform mixed-method analysis of feasibility data. This will inform final TAND-SQ design.

##### Step 2: The App Phase I Pre-pilot Study

Each consortium member will conduct user acceptance testing of phase I of the app (i.e., all app functionality stated under App Development, excluding the ‘TAND toolkit'), and complete an app feedback form. Each consortium member will collate their additional feedback on the app, e.g., screenshots and specific comments, in a PowerPoint or Word document and submit this to the project coordinator. All feedback collected will be reviewed by the Technology Group, Family Representatives Group and Action Group, either for immediate implementation or for future ‘scale-up and scale-out' efforts (which fall beyond the scope of this current project). All feedback related to the assurance of data quality and integrity and optimal user experience will be implemented in the app before data collection step 3 commences to ensure that the app is built for accurate data collection and optimal user experience.

##### Step 3: The TAND-SQ Validation Study

Data collection from BCH and CCH participants will take place via the app, they will complete a TSC Story, TAND-SQ Checklist and an app feedback form. Pseudonymised app data will be linked to de-identified detailed phenotyping data collected at BCH/CCH as part of an ongoing rare diseases study (21). The de-identified data will encompass a broad range of phenotyping data including (but not limited to) standardised rating scale measures e.g., the Behavior Rating Inventory of Executive Function (BRIEF) (22, 23), and the Child Behavior Checklist (CBCL) (24, 25), formal Intelligence Quotient (IQ), neuropsychological and scholastic measures, as well as standardised clinical diagnostic tools e.g., the Autism Diagnostic Observation Schedule Second Edition (ADOS-2) (26, 27), and the Autism Diagnostic Interview Revised (ADI-R) (28).

Participants from the TSC Alliance will complete a TAND-SQ Checklist through a secure electronic portal. TAND-SQ data collected through the TSC Alliance portal will be linked to real-world clinical TAND data which is also collected through the portal as part of an ongoing longitudinal natural history study (29). Data will be shared with the study team through a secure electronic portal.

Data will be analysed to establish the external and predictive validity of the TAND-SQ in relation to two different datasets—one a ‘real-world' clinical dataset, and the other a highly standardised research dataset. We plan to use expert data to code participants for each natural cluster (both categorically and in a quantified form) blind to the TAND-SQ data. We will then compare these data (deep phenotyping and clinic-derived) to the automated classifications from the TAND Toolkit App. In addition, we will examine external validity by comparing TAND-SQ cluster domain scores with specific instrument scores such as the BRIEF, CBCL and so on. The key question that we want to answer is whether or not the TAND-SQ Checklist is sensitive enough to identify TAND difficulties that had been picked up by expert evaluation. There is less of a priority on specificity, given that this is a *screening* tool (to lead to next-step actions) rather than a *diagnostic* tool.

Feedback form data will be analysed using descriptive and summative analysis to examine the clarity, comprehensiveness and ease of use of the app. These data may indicate user-interface or technical issues that may require further changes to be made to the app before continuing with step 4, App Phase II Pre-pilot Study.

All TAND-SQ data will be used to replicate and expand previous cluster work. We will apply the same clustering analysis technique developed by Leclezio et al. (13), de Vries et al. (14), and Leclezio (15) to explore the natural TAND clusters identified through TAND-SQ data. This will allow an opportunity to compare natural clusters derived from TAND-L data to TAND-SQ data. In addition, TAND-SQ data will be used to explore how quantified cluster data could be utilised in the app and in future research.

##### Step 4: The App Phase II Pre-pilot Study

Each consortium member will conduct user acceptance testing of phase II of the app (i.e., the ‘TAND toolkit') and complete a toolkit feedback form. Each consortium member will collate their feedback on the ‘TAND toolkit,' e.g., screenshots and specific comments, in a PowerPoint or Word document and submit this to the project coordinator. The toolkit feedback form will be completed via a secure online portal. All feedback collected will be used to inform final app design, and will be reviewed and prioritised by the Technology Group, Family Representatives Group and Action Group, for either immediate implementation or prioritisation for future ‘scale-up and scale-out' efforts. Changes requiring immediate implementation will be completed before proceeding with step 5, feasibility evaluation.

##### Step 5: The App Feasibility Evaluation Study

Each participant will conduct user acceptance testing of the full app (phase I and II), complete an app feedback form and toolkit feedback form. Each participant will also participate in a focus group / semi-structured interview, either in person or via a remote communication tool. Simple descriptive statistics will be used to summarise the quantitative comments, and thematic analysis will be used to summarise the qualitative comments. See Leclezio et al. (11) for detail of this methodology. The results from this feedback will be used to prepare ‘scale-up and scale-out' recommendations for future improvement and implementation of the TAND-SQ Checklist and the TAND Toolkit App. The survey functionality in the app will also be used in future to get feedback from families, allowing us to learn more about TAND. It would be possible to add a short survey to, for instance, ask families to rate and provide feedback on specific toolkit recommendations.

### Data Protection, Management, Storage, and Transfer

Given that this is a multisite project, with data collection sites located in the USA and Belgium, and data processing sites located in Belgium and South Africa, various data privacy regulations need to be considered: the Health Insurance Portability and Accountability Act (HIPAA) (30) and the Children's Online Privacy Protection Act (COPPA) (31) of the USA, the General Data Protection Regulation (GDPR) (32) of the European Union, and the Protection of Personal Information Act (POPIA) (33) of South Africa.

At present, the GDPR is seen as the most stringent standard for data protection. All data collection, protection, management, storage and transfer will be handled in strict compliance with the GDPR (32). The TAND Toolkit App will collect limited personal data, such as first name, year of birth, sex, personal pronouns and country. The app will not collect any personal identifiers as defined by HIPAA (30), such as full name, date of birth, address, email address, mobile contact numbers or device serial numbers. In accordance with HIPAA (30), the limited personal data that the app will collect does not meet criteria for identifiable personal health information—the app is therefore exempt from HIPAA. COPPA relates to online data collection directly from minors under the age of 13 years (31). In the TANDem study, no children under the age of 13 years will use the TAND Toolkit App themselves, their families/caregivers will be invited to participate in the study and complete the app about their child; COPPA thus does not apply. POPIA is also not applicable as we will not be collecting any data from participants in South Africa (33).

During the course of the 4-year project we will continue to ensure that the app and the research it enables remain compliant with the highest level of data protection and security, in accordance with the GDPR. Informed consent is the legal basis for collecting, sharing and processing data. Risks of reidentification of individuals are mitigated through several processes. Pseudonymised data will be collected and transferred between the data providers and data processors. A unique user code will be assigned to each app user by the respective data collection sites (data providers). This user code will be used as the username, along with a user-chosen password, to log in to the TAND Toolkit App. Data processors will use this unique user code to link app data with expert clinical data for data analysis purposes, as described in step 3—the TAND-SQ Validation Study. A master key with identifiable information will be kept at the primary data collection sites. No personally identifiable information will be shared by the data providers. Data will be shared with the data processors through a secure electronic portal. All participating data collection sites (data providers) and recipient sites (data controllers and data processors) will sign a data transfer agreement. All researchers participating in data collection will have completed Good Clinical Practice training and will be bound by the clinical rules of ethics, including confidentiality of patient information.

The app itself will also be designed for GDPR compliance. It will be password protected and all necessary encryption protocols will be implemented by the app developers. Users will have complete control over and access to their own data, and will be able to delete their data from their mobile device and the data storage server. An electronic informed consent, privacy policy and terms of use will be developed for the app. The electronic informed consent is a requirement for both research ethics and GDPR, it describes the study objectives and expected outcomes, the risks and benefits of participating in the research, the option to withdraw consent at any time, and other details. The privacy policy stipulates how personal data is protected, what types of data are collected in the app, who are the data controllers, how the data will be processed and stored, and what the rights of the app users are with respect to the privacy of their data, etc. The terms of use is the agreement between the app user and the app owner, and explains what the app does, who owns which app content, how the content can be used by the app user and by the app owner, etc. Each participant or consortium member will be required to read and accept the electronic informed consent, privacy policy, and terms of use (accessed in the app) before they will be allowed to register as app users in the TAND Toolkit App. All app data will be hosted on GDPR compliant servers, and will be stored securely and indefinitely. All other research data will be stored securely for 10 years, or as per the ethics guidelines of the respective data collection sites.

Table 4 lists some important considerations when developing a GDPR compliant app for research purposes. Please note that this is not an exhaustive list of all requirements. The local research ethics committee and data protection officer will be able to provide guidance with conducting a data protection impact assessment and developing a data management plan. A data protection officer and a lawyer specialised in data protection legislation will be able to provide guidance with developing the data sharing agreement and the data processing agreement. The data management plan outlines the full life-cycle of the data, who the data collectors/providers are, who the data recipients/processors are, details on how the data will be managed, transferred and stored securely, what risks are involved, and how these risks will be mitigated. A data sharing agreement stipulates the purpose of data sharing, how the data will be used, what data will be shared, who the data providers, data controllers and data processors are, and what their respective data protection responsibilities are. The data processing agreement stipulates the scope and purpose for which the data will be used and how the data will be processed, as well as the data protection responsibilities for each data controller and data processor involved. These are legally binding agreements that need to be signed by all parties involved in the study.

**Table 4:** Considerations when developing a GDPR compliant app for research purposes.

1.	Conduct a data protection impact assessment to ensure that all risks are identified, assessed and mitigated
2.	Determine which data protection regulations are applicable based on the locations of all data collection and data processing sites involved
3.	Create a data management plan
4.	Create a data sharing agreement
5.	Create a data processing agreement
6.	Search for app developers who have expertise in developing and hosting apps in compliance with the GDPR
7.	Sign a non-disclosure agreement with the app developers and a contract that ensures that one retains ownership of the app, intellectual property and data
8.	Develop the app on an open-source platform rather than a proprietary/exclusive platform; this will allow one to more easily transfer the app development/support to another service provider in future should it be required
9.	Develop an electronic informed consent for the app; in the app the app user must be able to view and download/print this document
10.	Develop a privacy policy for the app; in the app the app user must be able to view and download/print this document
11.	Develop terms of use for the app; in the app the app user must be able to view and download/print this document
12.	The first step in the app is to have prospective app users read and agree to the electronic informed consent, privacy policy, and terms of use. Only once app users have provided this consent can any app user registration and other data be captured
13.	The app should be password protected
14.	The app and data administration panel must implement the necessary encryption protocols and strategies for data protection and security
15.	App users must be able to control, access and delete all of their own app data on the mobile device and the storage servers
16.	Multifactor authentication must be implemented for all means of accessing captured/stored data via the app data administration panel or secure cloud storage solution
17.	Ensure that the location of the app hosting servers and the applicable data protection legislation in that country/state/region meet the requirements for GDPR compliance
18.	Retain separate staging (testing) and production environments of the app data administration panel, this way all ongoing iterative development and testing can be done in the staging environment without affecting the app and live data in the production environment
19.	Ensure that only the data controllers/processors have access to the live (real person) data in the production environment. If support is required from an app developer, server manager, or other third party, ensure that a sufficient data processing agreement has been signed by all parties involved

### Ethical Considerations

#### Participant Vulnerability

The TANDem project will include participants who may be considered to be of medium vulnerability. This includes adults with TSC (a rare genetic disease), adults with some degree of intellectual or developmental disability and young people 13–18 years of age. As outlined in this paper, all research will be led through expert TSC centres and organisations and all research recruitment will be done through these organisations. We are therefore confident that participants and families will be invited and selected in a manner sensitive to their level of potential vulnerability. In the spirit of distributive justice, it was important not to exclude young people and adults with developmental disabilities from this study, as they represent a fundamentally important stakeholder group in this study. For adults with more significant disabilities and children under the age of 13, we will recruit parents/caregivers to participate on their behalf.

#### Participant Risks

Given that all the research work will be qualitative and participatory, this project meets criteria to be regarded as a minimal risk study. As outlined in Table 3, participants will be asked to complete a paper TAND-SQ Checklist and checklist feedback form, and/or complete the TAND Toolkit App (capture a TSC Story, TAND-SQ Checklist, and an app feedback form), and/or complete the TAND-SQ via an online portal, and/or complete a toolkit feedback form, and/or participate in a focus group / semi-structured interview. Participants will also give permission for their retrospective medical records to be accessed and pseudonymised data to be shared for the analysis goals of this study. Importantly, the information in the app will also be pseudonymised on the app storage servers and will not collect any personal identifiers.

No interventional procedures will be performed. No children below the age of 13 will be included as direct project participants. Therapeutic misconception will be avoided at recruitment, because the purpose of the study will be made clear to families on recruitment and in the terms of use of the app. No other prospective evaluations or interventions will be performed.

#### Informed Consent

All consortium members and participants will be asked to provide informed consent before data collection. Consortium members will be provided with information sheets and informed consent forms by the postdoctoral researcher. Requesting informed consent from consortium members signals the participatory nature of this project, thus allowing, for example, consensus discussions to be recorded, collected and analysed in a systematic way as ‘data' of this study.

At the clinical research sites (BCH, CCH, UZB, and UZL), individuals with TSC and/or their families/caregivers will be invited by the TSC clinic team to participate. The objectives, study set-up and expected outcomes of the project will be explained in detail to each prospective participant guided by the informed consent document. Individuals will be given time to think about whether or not they would like to participate, and to discuss the matter with friends/relatives. At any time, individuals with TSC and family members can ask questions or make comments.

Informed consent documents will be available in the native languages of the participants to be enrolled. The USA sites will conduct work in English. For the Belgian sites, the informed consent documents will be available in English and Dutch. During this project, the TAND Toolkit App and all feedback forms will only be available in English. Qualitative interviews will, however, be conducted either in English or Dutch, based on the preference of participants. Participants will be able to withdraw their consent at any stage. Parents/caregivers and individuals over 18 years will be asked for written informed consent. Individuals between the ages of 13 and 18 years will be asked for written assent with support from their parents/caregivers.

Individuals completing the app will also be required to provide electronic consent that they have read and understood the electronic informed consent, privacy policy, and terms of use documents within the app before being allowed to register as app users.

For data collection taking place through the TSC Alliance electronic portal, individuals will provide informed consent via the portal. Families/Caregivers of already consented participants of the TSC Alliance database will receive individual email invitations to participate. They will be able to complete the TAND-SQ Checklist online through the portal, and data will automatically be captured and saved to the TSC Alliance database.

## Discussion

### In Need of Multidisciplinary Phenotyping and Treatment Design for Psychopathological and Neurocognitive Disorders in Genetic Syndromes

There are many thousands of rare genetic diseases where significant progress has been seen in recent decades in understanding of the molecular pathways in many syndromes, and in the identification and treatment of the physical phenotypes/manifestations associated with these syndromes. However, many genetic syndromes are also associated with a wide range of behavioural, psychiatric, intellectual, academic, neuropsychological and psychosocial difficulties and disorders. In the majority of syndromes these are highly under-identified and under-treated. This manuscript forms part of a special issue on the need of multidisciplinary phenotyping and treatment design for neuropsychiatric disorders in genetic syndromes—and the rationale for this pressing need is clear.

In this protocol paper, we focused on Tuberous Sclerosis Complex (TSC) and TSC-associated neuropsychiatric disorders (TAND) as example. Through participatory research with family stakeholders in the TSC community, we were able to identify priority areas to empower families and people living with TSC towards improved (self-)phenotyping, and to use technology as a tool to direct them to educational information, links and resources that would ultimately support patient/individual-centred health care. This is the primary aim of the TANDem project. Recognising the limited ‘evidence-base' for interventions in TSC (and other genetic syndromes), our second aim is to generate consensus clinical recommendations for identification and intervention of TAND manifestations. We hope that this will also empower clinicians with up-to-date knowledge and information to support their clinical decision-making, which we acknowledge can be very complex in the context of rare genetic syndromes. Recognising the need for a ‘next generation' of TAND researchers and for expanded knowledge and awareness about TAND in the broader community, our third aim is to have a strong focus on research capacity-building, public engagement and creation of a digital infrastructure for future research.

### Impact on the TSC Community

The TANDem project has the potential to transform the landscape for TAND identification and intervention at a global level. A well-designed app can be made accessible internationally to empower families with relevant and up-to-date knowledge about TSC and TAND. An app provides the opportunity to translate information into various languages in future scale-out, and the toolkit can be updated as new evidence becomes available. Even without the technological elements of the project, a self-report, quantified TAND Checklist will provide a validated tool to support families in profiling, monitoring and intervening with TAND, while additional help is sought. Given that the burden of TAND is the most pronounced aspect of TSC and given the priority of interventions for TAND, empowering families with self-help tools, skills and information around TAND will have a major impact on their quality of life, quality of care and social participation. This project is designed in partnership with family stakeholders and a wide range of other stakeholders to ensure that the impact of the research will be direct, immediate, ongoing and relevant.

**Figure 4:**
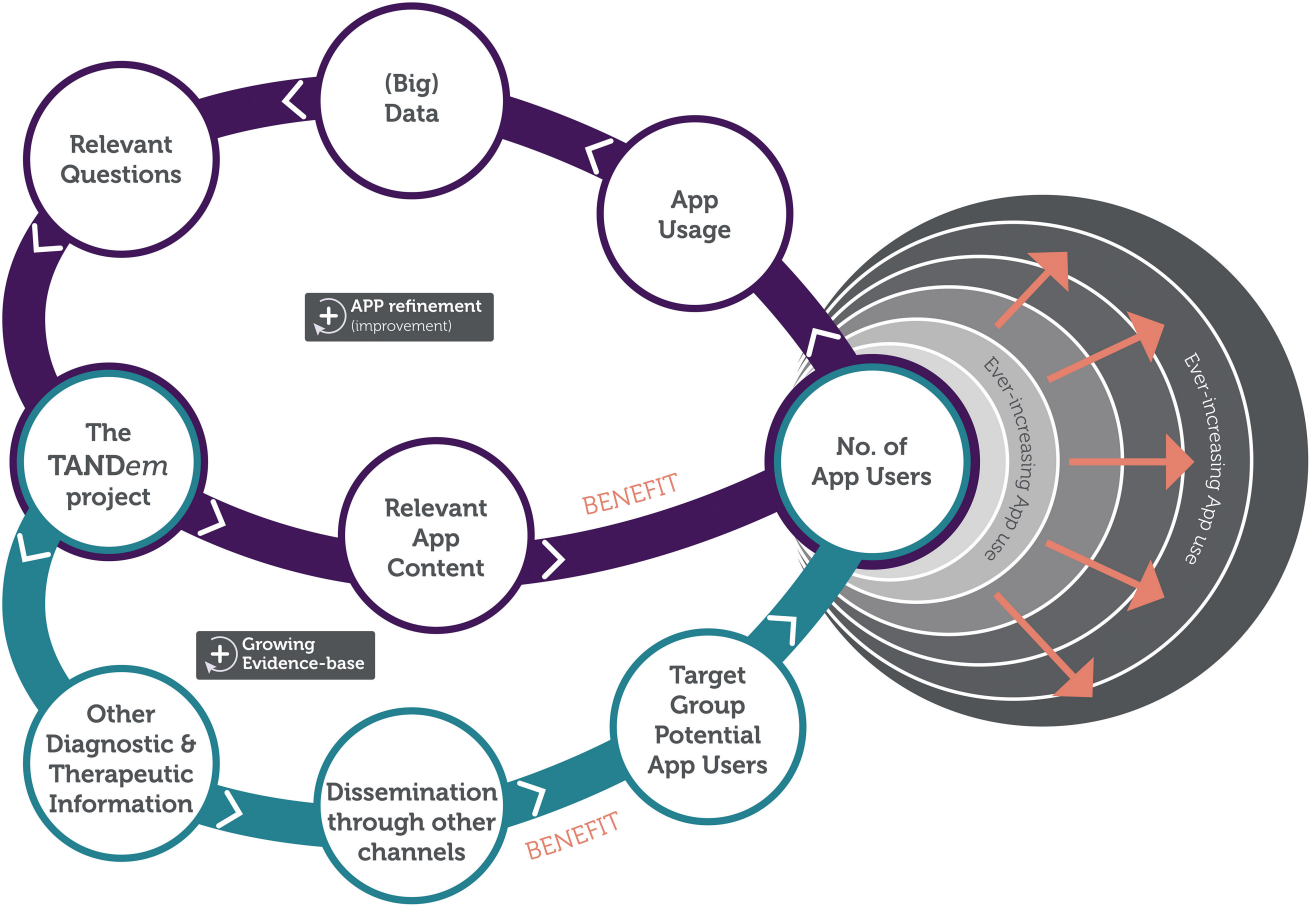
TANDem project impact loop.

Apart from the direct benefits to individuals with TSC and their families, the project will create a digital infrastructure to collect big data that will allow for further optimisation of the TAND toolkit, for better TAND management for individuals with TSC and their families and for fueling global TAND research in the future. The digital technology, as will be developed in the TANDem project, will therefore create the potential for large-scale (and self-guided) phenotyping, that in turn can inform large-scale (and self-guided) intervention planning. Figure 4 shows the impact loop of the overall study.

### Future of the App

The overarching goal of this project is to develop an app for the TSC community with a good evidence-base and with accurate self-help information and consensus recommendations for the identification and intervention of TAND. During the 4 years of the project, the app will be used purely within the TAND consortium and will not be available to access by the public. One of the goals of the project in year 4 is to prepare for scale-up and scale-out of the app. This will include separate conversations about data curation, access, security, servers etc. The philosophy of the app is to make the tool freely accessible in as many languages as possible and to aim for global reach. Families/Caregivers/App users should never have to pay for the app, and we will ensure that the app is never used for any commercial purpose. This will be achieved (a) through a creative-commons license on materials, (b) through a non-disclosure contractual agreement with the app developers and (c) through development of the app on an open-source platform. At the end of the project, the app developers will hand over the full app to the TAND consortium who will be the ‘owners' of the app. The TAND consortium will therefore be able to revisit and renegotiate contracts with the app developers or may move to other developers. This way the TSC community will retain control of the data collected, and app companies will only be used to support our goals rather than to have any ownership of TAND data.

### How We Will Use the Data in Future

There are many potential uses of the data to be collected through the TAND Toolkit App. These could include refining and updating natural TAND clusters, collecting of preliminary data on new research ideas via the survey functionality in the app, informing app users of potential research studies for participation, and considering how and if the TAND-SQ could be a useful patient/self-reported outcome measure for future clinical trials of pharmacological or non-pharmacological interventions of TAND. Other research uses could include studying the natural history of TAND over the lifespan, or to assess response to TAND-focused interventions. On completion of the TANDem project, a clear data management and curation plan will have been drawn up and clear procedures will be put in place for application from internal or external researchers to apply for access to specific data to answer specific research questions. We will want to ensure that data access and future research using TAND Toolkit App data will be done in line with the spirit and philosophy of the overall TANDem project, including participatory principles, direct benefit of research to the community we serve, and socially responsive research. One of the fundamental principles which is easily managed through an app is the recognition that individuals and families are the ultimate ‘owners' of their own data, and that they can at any point decide to remove their data from the app.

### Are We Developing a Medical Device or a Health App?

There has been growing interest in the digital health literature about health apps vs. medical devices. Different countries and jurisdictions are developing their own sets of guidelines and regulations about medical devices, including definitions of medical devices. The USA Federal Drug Administration provided a very helpful list of mobile apps that are not medical devices (34). The TAND Toolkit App represents an app for general education and to facilitate access to reference information to those in the TSC community. All information provided in the app (TAND-SQ Checklist, clusters, consensus recommendations, etc.) will be published in the peer-reviewed scientific literature, and will be presented in the app in a user-accessible format. The app will filter information to people with specific characteristics (e.g., specific natural TAND clusters) in order to provide patient/individual awareness, education and empowerment. The app will, however, at no stage perform any diagnostic procedures or diagnostic analytics, provide any direct treatment or replace any clinician decision-making. We are therefore clear that the TANDem project aims to develop a health app but not a medical device. Ultimately, as outlined in the background section of this protocol paper, we aim to reduce the identification and treatment gap for TAND by empowering families with a digital tool that will provide them with a portal to access accurate information, help their own health decision-making in terms of what to seek as next step professional support, and by providing them with general and practical tips of things they can do at home to improve their journey with TSC and TAND.

## Author Contributions

TH, ACJ, and PJdV drafted the manuscript. All authors contributed to the design, drafting, revising of the TANDem study protocol, critically reviewed and revised the manuscript, and approved the final manuscript prior to submission.

## Funding

This work was funded by a grant from the King Baudouin Foundation Fund Dr. & Mrs. Charles Tournay-Dubisson to PJdV and ACJ (2019-J1120010-213544) and supplemental funding from the Tuberous Sclerosis Association (UK) (2019-P03).

## Conflict of Interest

SB is funded by Cerebra to investigate sleep and behaviour in rare genetic syndromes, including TSC. PD receives partial salary support from Aucta Pharmaceuticals for a study of topical sirolimus for facial angiofibromas in TSC and Marinus Pharmaceuticals for a study of ganaxolone for TSC-related epilepsy. ACJ was on the scientific advisory group of the TOSCA international disease registry sponsored by Novartis. DK reports personal fees from Novartis Pharmaceuticals, personal fees from Greenwich Bioscience, grants from Marinus Pharmaceuticals, personal fees from Nobelpharma America, and personal fees from REGENXBIO outside the submitted work. MS reports grant support from Novartis, Biogen, Astellas, Aeovian, Bridgebio, and Aucta; and has served on Scientific Advisory Boards for Novartis, Roche, Regenxbio, SpringWorks Therapeutics, Jaguar Therapeutics, and Alkermes. CS receives salary support from GW Pharma, Mallinckrodt, Nobelpharma, Novartis, Ovid, UCB, and Upsher-Smith. PJdV was a study steering committee member of three phase III trials sponsored by Novartis and on the scientific advisory group of the TOSCA international disease registry sponsored by Novartis. AMvE reports a grant from GW Pharmaceuticals for TAND-related research during the conduct of the study. The remaining authors declare that the research was conducted in the absence of any commercial or financial relationships that could be construed as a potential conflict of interest. The handling editor declared a shared affiliation, though no other collaboration, with one of the authors SB at the time of review.

## Publisher's Note

All claims expressed in this article are solely those of the authors and do not necessarily represent those of their affiliated organizations, or those of the publisher, the editors and the reviewers. Any product that may be evaluated in this article, or claim that may be made by its manufacturer, is not guaranteed or endorsed by the publisher.

## Abbreviations

ADOS-2: autism diagnostic observation schedule second edition
ADI-R: autism diagnostic interview revised
BCH: Boston Children's Hospital
BRIEF: behavior rating inventory of executive function
CBCL: child behavior checklist
CCH: Cincinnati Children's Hospital
COPPA: children's online privacy protection act
GDPR: general data protection regulation
GRADE: grading of recommendations assessment, development and evaluation
HIPAA: health insurance portability and accountability act
IQ: intelligence quotient
POPIA: protection of personal information act
TSC: tuberous sclerosis complex
TAND: TSC-associated neuropsychiatric disorders
TAND-L: TAND checklist (lifetime version)
TAND-SQ: self-report, quantified TAND checklist
UZB: Universitair Ziekenhuis Brussel
UZL: Universitaire Ziekenhuizen Leuven.
